# Interrelation among one-carbon metabolic (OCM) pathway-related indicators and its impact on the occurence of pregnancy-induced hypertension disease in pregnant women supplemented with folate and vitamin B12: Real-world data analysis

**DOI:** 10.3389/fnut.2022.950014

**Published:** 2023-01-10

**Authors:** Yanfei Zhang, Chenhong Gu, Ying Lei, Jingwen Wang, Leiqin Sun, Junwei Fan, Yanlin Wang, Xiaoqing Zhang

**Affiliations:** ^1^Department of Pharmacy, The International Peace Maternity and Child Health Hospital, School of Medicine, Shanghai Jiao Tong University, Shanghai, China; ^2^Shanghai Key Laboratory of Embryo Original Diseases, Shanghai, China; ^3^Department of General Surgery, Shanghai General Hospital, School of Medicine, Shanghai Jiao Tong University, Shanghai, China; ^4^Division of Maternal-Fetal Fetal Medicine, Prenatal Diagnosis Center, The International Peace Maternity and Child Health Hospital, School of Medicine, Shanghai Jiao Tong University, Shanghai, China

**Keywords:** one-carbon metabolism, folate, vitamin B12, homocysteine, methylenetet-rahydrofolate reductase, pregnancy-induced hypertension

## Abstract

**Background and objective:**

A considerable number of pregnant women who were supplemented with folate and vitamin B12 were selected as major participants in studying the one-carbon metabolic (OCM) pathway. Our study aimed to explore the effects of OCM-related indicators on pregnancy-induced hypertension (PIH) and preeclampsia (PE) in pregnant women with folate and vitamin B12 supplementation.

**Subjects and methods:**

A total of 1,178 pregnant women who took multivitamin tablets containing 800 μg folate and 4 μg vitamin B12 daily from 3 months before pregnancy to 3 months after pregnancy were enrolled in this study. These pregnant women were classified into three groups: the normotensive group (*n* = 1,006), the PIH group (*n* = 131), and the PE group (*n* = 41). The information on age, weight, body mass index (BMI), number of embryos, gravidity, parity, and OCM-related indicators (serum level of homocysteine, folate, and vitamin B12; MTHFR C677T genotype) was collected.

**Results:**

The accuracy of the prediction model based on the screened independent risk factors (hyperhomocysteine, OR = 1.170, 95% CI = 1.061–1.291; high folate status, OR = 1.018, 95% CI = 0.999–1.038; and high BMI, OR = 1.216, 95% CI = 1.140–1.297) for PIH in subjects with MTHFR CC genotype (AUC = 0.802) was obviously higher than that in subjects with MTHFR CT, TT genotype (AUC = 0.684,0.685, respectively) by receiver operating characteristic curve analysis. The homocysteine level of the PIH group was significantly higher than that of the normotensive group only in subjects with the MTHFR CC genotype (*p* = 0.005). A negative correlation between homocysteine and folate appeared in subjects with MTHFR CT + TT genotype (*p* = 0.005). A model including multiple embryos, nulliparas, and lower folate could predict the process from PIH to PE (AUC = 0.781, *p* < 0.0001).

**Conclusion:**

The prediction model composed of homocysteine, folate, and BMI for PIH was suitable for subjects with MTHFR CC genotype in pregnant women with supplementation of folate and vitamin B12. Lower folate levels could be an independent risk factor in developing the process from PIH to PE.

## Introduction

More than 10% of pregnant women suffered from hypertensive disorders due to pregnancy (HDP). The incidence of HDP has increased by 20% in the recent 20 years caused by the increases in obesity and pregnancy comorbidities, such as gestational diabetes and hyperlipidemia. Although diagnostic criteria of hypertension in the general population was revised by the American College of Cardiology/American Heart Association in 2017, pregnancy-induced hypertension (PIH) was still defined as systolic blood pressure (SBP) ≥140 mmHg and/or diastolic blood pressure (DBP) ≥90 mmHg, measured on two separate occasions. Approximately 25 to 50% of pregnant women with PIH would eventually develop into preeclampsia (PE). The classic triad of signs and symptoms of PE were hypertension, edema, and proteinuria, which were also used as the diagnostic bases of PE (1, 2).

Hypertensive disorders due to pregnancy might cause serious consequences for both the pregnant woman and the fetus. Pregnant women with HDP have a higher incidence of cesarean, premature birth, low-birth-weight infant, admission to neonatal ward, and newborn perinatal death. HDP was associated with increased maternal morbidity and mortality, predominantly because of maternal stroke and increased risk of developing cardiovascular disease (CVD) in pregnant women later in life. Multiple hormonal, vascular, and metabolic mechanisms had been proposed to explain the pathological process of hypertensive diseases during pregnancy. The most studied risk factors of HDP included mothers greater than 35 years of age, obesity before pregnancy, excess weight gain during pregnancy, gestational diabetes, family history of hypertension, and genetics. However, the accuracy of these HDP risk factors remained in doubt, and there was still a lack of an effective model for predicting HDP (3).

Homocysteine (HCY), a nonessential amino acid containing sulfur, had been proven to play an important role in the pathophysiology process of hypertension. The demethylation of methionine produced homocysteine. Hyperhomocysteinemia was caused by various environmental factors which induced a deficiency of folate, vitamin B12, and vitamin B6. It had been reported that hyperhomocysteinemia was related to 24-h systolic and diastolic blood pressure values. Mechanisms of the contribution of hyperhomocysteinemia to the development of hypertension could include the stimulation of smooth muscle cell proliferation, injury of vascular endothelium, inducing vascular oxidative stress, and reducing vascular elastic compliance by lowering the ratio of the vascular elastin/collagen (4). There is mounting evidence that hyperhomocysteinemia was involved in this pathological process of essential hypertension. Also, hyperhomocysteinemia was gradually recognized as a predictive marker for PIH and PE occurrence (5, 6).

Homocysteine was an important intermediate product in the one-carbon metabolic (OCM) pathway and other participants in this pathway could influence the circulating concentration of homocysteine (7, 8). 5-Methyltetrahydrofolate (5-MTHF) was the major form of folate in the human body and served as a substrate in the metabolic transformation of homocysteine to methionine *via* methionine synthase (MTR). Methionine is converted to S-adenosylmethionine (SAM) in the liver by methionine adenosyltransferase (MAT). SAM supplied methyl donors for methylation that played a pivotal role in biochemical reactions involved in multiple pathophysiological processes. Vitamin B12 took part in the re-methylation process of homocysteine to methionine catalyzed by MTR as an intermediate methyl carrier. Suitable intake and serum concentration of folate and vitamin B12 were necessary to maintain the normal concentration of homocysteine (9, 10).

Genetic polymorphisms of metabolizing enzymes related to the OCM pathway might impact the conversion of homocysteine to methionine and be associated with circulating homocysteine concentrations. Methylenetetrahydrofolate reductase (MTHFR) was the key enzyme of OCM and converted 5,10- methylenetetrahydrofolate (5,10-CH2-THF) to 5-MTHF, which was the active form of folate and participated in the remethylation of homocysteine to methionine as a methyl donor (11, 12). MTHFR C677T polymorphism was a functional site, which was associated with the incidence of PIH and PE in many previous studies (13–15).

The low folate level in pregnancy was due to increased requirements, insufficient folic acid intake, and increased loss. In obstetric clinical practice, a considerable number of pregnant women were supplemented with folate and vitamin B12 using compound vitamin supplements aimed to prevent maternal and fetal complications such as anemia, abortion, premature delivery, and fetal neural tube malformation. The effects of folate and vitamin B12 supplementation on interrelation among OCM-related indicators remained unclear. This study aimed to explore the impact of OCM indicators on PIH and PE in Chinese Han women with folate and vitamin B12 supplementation by real-world data (RWD) analysis.

## Subjects and methods

### Study design and participants

Pregnant women who underwent physical examination, and genotyping of MTHFR C677T and gave birth in the International Peace Maternity and Child Health Hospital (IPMCH) of China welfare institute from June 2018 to August 2021 were included in this study. All pregnant women took multivitamin tablets (Menevit, Bayer company, Leverkusen, Germany) containing 800 μg folate and 4 μg vitamin B12 daily from 3 months before pregnancy to 3 months after pregnancy. At present, multiple varieties of multivitamin drugs were used clinically. However, the contents of various vitamins in these drugs are different which could lead to confused and difficult to interpret test results. Therefore, all the pregnant women took the same multivitamin tablet contained 800 μg folate and 4 μg vitamin B12 daily were selected as the subjects of this study. The case-control study design was applied using real-world data (RWD) of all subjects meeting the inclusion criteria and exclusion criteria: 85.40% (*n* = 1,006) of cases had normal blood pressure; 11.12% (*n* = 131) of cases had PIH; 3.48% (*n* = 41) of cases had PE. All participants signed an informed consent form.

Pregnancy-induced hypertension was defined as systolic blood pressure (SBP) ≥140 mmHg and/or a diastolic blood pressure (DBP) ≥90 mmHg after 20 weeks of gestation, which returned to normal 12 weeks after delivery; proteinuria was negative. PE was defined as systolic blood pressure (SBP) ≥140 mmHg and/or a diastolic blood pressure (DBP) ≥90 mmHg after 20 weeks of gestation; 24-h proteinuria was more than 0.3 g/day or random proteinuria was positive. When proteinuria was negative, any of the following occurred: decreased platelet count (<100 × 10^9^/L); liver function damage (serum transaminase level more than two times the upper limit of normal); impaired renal function (serum creatinine level more than two times the upper limit of normal, or beyond 1.1 mg/dL); pulmonary edema; new occurrence of central nervous system abnormalities or visual impairment.

The inclusion criteria were as follows:

(1) Women of childbearing age of over 18 years.
(2) Genetic testing of MTHFR 677TT polymorphism.
(3) Folate and vitamin B12 supplementation was carried out.
(4) Serum levels of vitamin B12, folate, and homocysteine were detected at 10–14 weeks of gestation.
(5) The whole process of prenatal examination, including blood pressure monitoring, was performed in IPMCH.

Exclusive criteria:

(1) Pre-existing hypertension before pregnancy.
(2) Secondary hypertension: hypertension caused by kidney disease, aldosteronism, pheochromocytoma, etc.
(3) History of chronic kidney disease.
(4) History of dysfunction of important organs such as the heart, lungs, liver, and kidney.
(5) Severe anemia (hemoglobin <60 g/L).
(6) Pregnant women with malignant tumors.
(7) Serious infections.

## Data collection

One-carbon metabolic-related indicators, including serum levels of vitamin B12, folate, homocysteine, and MTHFR C677T genotype were retrieved and sorted out from the maternal database of IPMCH. Clinical parameters of enrolled subjects were collected, including age, weight, body mass index (BMI), number of embryos, gravidity, and parity.

Serological index detection: The timing of sampling of serum level of homocysteine, folate, and vitamin B12 was 3 months after pregnancy. PIH was defined as systolic blood pressure (SBP) ≥140 mmHg and/or diastolic blood pressure (DBP) ≥90 mmHg after 20 weeks (5 months) after pregnancy. The present study explored whether these indicators can be used as predictive risk factors for PIH. Serum homocysteine level was detected using the Beckman Coulter automatic biochemical analyzer (Beckman Coulter, Inc., Brea, CA, USA). Serum vitamin B12 and folate levels were determined with the chemiluminescence method using an ARCHITECT C16000 automatic biochemical analyzer (Abbott Laboratories, Chicago, IL, USA).

Genotyping of MTHFR C677T polymorphism: A total of 2 ml of human peripheral blood samples were collected and put into an EDTA-treated anticoagulant tube. Samples were genotyped according to the protocol of the MTHFR (C677T) gene detection kit (Shanghai BaiO Technology Co., Ltd., Shanghai, China).

## Data analysis

SPSS 26.0 software package (IBM Corporation, Armonk, NY, USA) and Graphpad prism8 (GraphPad Software, San Diego, CA, USA) were used for statistical analysis and mapping. Measurement data were expressed as $\bar{\text{x}}$ ± s. The groups were compared using a *t*-test or One-way analysis of variance (ANOVA). The counting data were expressed in terms of frequency and percentage. χ^2^ test was used to compare ratios between groups. Risk factors were analyzed by binary logistic linear regression analysis. Receiver operating characteristic (ROC) curve analysis was utilized to evaluate the accuracy of the prediction model. Pearson correlation analysis was used to analyze the relationship between variables. For all analyses, two-sided *p*-values of ≤0.05 were considered significant.

## Results

### Description of each parameter of OCM

For all the subjects, serum folate concentration was 37.34 ± 11.11 nmol/L, which was a normal high value (7.0–46.4 nmol/L); serum vitamin B12 concentration was 389.8 ± 165.5 pmol/L, which was normal median value (138–652 pmol/L); serum homocysteine concentration was 4.315 ± 1.623 μmol/L, which was normal low value (0–15 μmol/L). The frequency of MTHFR CC, CT, and TT genotypes was 30.22, 48.56, and 21.22%, respectively.

### Univariate analysis of factors influencing the incidence of PIH and PE

The weight and BMI of the PIH group and the PE group were significantly higher than those of the normotensive group, and there was no significant difference in weight and BMI between the PIH group and the PE group. There was no significant difference in age, number of embryos, gravidity, and parity among the three groups. Serum vitamin B12 concentration decreased in turn in the normotensive, PIH, and PE groups with a marginal statistical difference (*p* = 0.0540). The serum vitamin B12 concentration of the PE group was significantly lower than that of the normotensive group (*p* = 0.0323). The serum folate concentration in the PIH group was the highest, in the normotensive group, the serum folate concentration was higher and in the PE group, it was the lowest, and there were significant differences (*p* < 0.0001). There was a significant difference between serum homocysteine concentrations among the normotensive group, the PIH group, and the PE group (*p* = 0.0222). No significant associations were observed between the MTHFR C677T genotype and the incidence of PIH or PE (*p* = 0.9577, 0.8625, respectively) (Table 1).

**TABLE 1:** Comparison of general information, serum parameters, and MTHFR C677T genotype of three groups of research subjects.

*n*		Normotensive pregnancy group	Pregnancy-induced hypertension group	Preeclampsia group	*p*_*a*_ *	*p*_*b*_ *	*p*_*c*_ *	*p*_*d*_ *
		(*n* = 1,006)	(*n* = 131)	(*n* = 41)				
**Age**								
≥35	284	242 (24.06%)	36 (27.48%)	6 (14.63%)	0.2432	0.3887	0.1924	0.1435
<35	894	764 (75.94%)	95 (72.52%)	35 (85.37%)				
Weight (kilogram)		56.4 ± 7.8	61.4 ± 8.2	59.4 ± 7.4	<0.0001	<0.0001	0.0145	0.2003
Body mass index (BMI)		21.2 ± 3.0	22.7 ± 3.8	22.5 ± 2.8	<0.0001	<0.0001	0.0081	0.624
**Number of embryos**								
1	1,082	925 (91.95%)	122 (93.13*%)	35 (85.37%)	0.2722	0.733	0.1437	0.1997
≥2	96	81 (8.05%)	9 (6.87%)	6 (14.63%)				
**Gravidity**								
1	389	336 (33.40%)	41 (31.30%)	12 (29.27%)	0.3393	0.8905	0.1148	0.2063
2	460	386 (38.37%)	52 (39.69%)	22 (53.66%)				
≥ 3	329	284 (28.23%)	38 (29.01%)	7 (17.07%)				
**Parity**								
0	968	829 (82.41%)	110 (83.97%)	29 (70.73%)	0.136	0.7146	0.0634	0.0709
≥ 1	210	177 (17.59%)	21 (16.03%)	12 (29.27%)				
Serum vitamin B12 concentration (pmol/L)		393.7 ± 159.7	376.2 ± 137.2	339.6 ± 121.8	0.054	0.2319	0.0323	0.1276
Serum folate concentration (nmol/L)		37.25 ± 10.73	39.92 ± 13.87	31.49 ± 7.374	<0.0001	0.0099	0.0007	0.0003
Serum homocysteine concentration (μmol/L)		4.261 ± 1.554	4.618 ± 2.077	4.668 ± 1.676	0.0222	0.0181	0.1001	0.8853
**MTHFR C677T genotype**								
CC	356	304 (30.22%)	38 (29.01%)	14 (34.15%)	0.9821	0.9577	0.8625	0.8216
CT	572	488 (48.51%)	65 (49.62%)	19 (46.34%)				
TT	250	214 (21.27%)	28 (21.37%)	8 (19.51%)				

* pa: Statistical difference among the three groups. pb: Statistical difference between normal pregnancy group and pregnancy induced hypertension group. pc: Statistical difference between normal pregnancy group and preeclampsia group; pd: Statistical difference between pregnancy-induced hypertension group and preeclampsia group.

### Establishment and internal validation of prediction model for PIH

Respective impact of 10 potential risk factors including age, weight, BMI, number of embryos, gravidity, parity, serum levels of vitamin B12, folate, homocysteine, and MTHFR C677T genotype, on the incidence of PIH were assessed by binary logistic linear regression analysis based on Enter method for the variable introduction. High Serum homocysteine level was a statistically significant risk factor for PIH (*p* = 0.002, OR = 1.169, 95% CI = 1.058–1.291), while BMI and serum folate concentration had marginal statistical differences as risk factors for PIH (*p* = 0.083, 0.054, respectively) (Table 2).

**TABLE 2:** Influence of general information and OCM-related indicators on PIH incidence based on logistic linear regression analysis*.

Variables		B	S.E.	Wald	*p*	Exp (B)	95.0% CI for EXP (B)	
							Lower	Upper
	Age	−0.05	0.03	2.715	0.099	0.951	0.896	1.01
All the subjects	Weight	0.036	0.025	2.052	0.152	1.037	0.987	1.09
(*n* = 1,137)	Body mass index	0.123	0.071	3.006	0.083	1.131	0.984	1.301
Normotensive group (*n* = 1,006)	Number of embryos	−0.452	0.457	0.978	0.323	0.636	0.26	1.559
PIH group (*n* = 131)	Gravidity	0.211	0.144	2.16	0.142	1.235	0.932	1.637
	Parity	−0.144	0.304	0.226	0.634	0.866	0.477	1.569
	MTHFR C677T genotype	0.083	0.145	0.327	0.567	1.087	0.817	1.445
	Serum vitamin B12 concentration (ng/L)	0	0.001	0.001	0.982	1	0.998	1.002
	Serum homocysteine concentration (μmol/L)	0.156	0.051	9.358	0.002	1.169	1.058	1.291
	Serum folate concentration (mmol/L)	0.019	0.01	3.703	0.054	1.019	1	1.039
	Constant	−6.828	1.48	21.278	0	0.001		

Serum homocysteine concentration (OR = 1.170, 95% CI = 1.061–1.291), serum folate concentration (OR = 1.018, 95% CI = 0.999–1.038), and BMI (OR = 1.216, 95% CI = 1.140–1.297) were screened out and formed into a prediction model of PIH by logistic linear regression analysis based on the Forward–Wald method for variable introduction (*p* < 0.001). Model internal validation was made in subjects with MTHFR CC, CT, and TT genotypes, respectively. BMI was a statistically significant variable in the three subgroups with different MTHFR C677T genotypes. However, serum homocysteine and folate concentration was the only statistically significant variable in the subgroup with the MTHFR CC genotype (Table 3).

**TABLE 3:** Establishment and internal validation of prediction model for pregnancy induced hypertension based on logistic linear regression analysis (*n* = 1,137).

Variables		B	S.E.	Wald	*p*	Exp (B)	95.0% CI for EXP (B)	
							Lower	Upper
All the subjects	Body mass index	0.196	0.033	35.529	0	1.216	1.14	1.297
(*n* = 1,137)*	Serum homocysteine concentration (μmol/L)	0.157	0.05	9.824	0.002	1.17	1.061	1.291
	Serum folate concentration (mmol/L)	0.018	0.01	3.548	0.06	1.018	0.999	1.038
	Constant	−7.796	0.899	75.15	0	0		
MTHFR C677TCC	Body mass index	0.182	0.054	11.408	0.001	1.2	1.079	1.333
Subgroup (*n* = 342)**	Serum homocysteine concentration (μmol/L)	0.294	0.096	9.329	0.002	1.342	1.111	1.621
	Serum folate concentration (mmol/L)	0.041	0.018	5.536	0.019	1.042	1.007	1.079
	Constant	−9.053	1.628	30.928	0	0		
MTHFR C677T CT	Body mass index	0.164	0.052	9.771	0.002	1.178	1.063	1.306
Subgroup (*n* = 553)**	Serum homocysteine concentration (μmol/L)	0.085	0.088	0.934	0.334	1.089	0.916	1.293
	Serum folate concentration (mmol/L)	0.02	0.015	1.761	0.185	1.02	0.991	1.05
	Constant	−6.833	1.328	26.485	0	0.001		
MTHFR C677T TT	Body mass index	0.174	0.072	5.906	0.015	1.19	1.034	1.369
Subgroup (*n* = 242)**	Serum homocysteine concentration (μmol/L)	0.062	0.1	0.384	0.535	1.064	0.875	1.294
	Serum folate concentration (mmol/L)	−0.014	0.021	0.458	0.499	0.986	0.947	1.027
	Constant	−5.491	1.975	7.729	0.005	0.004		

*Establishment of prediction model for pregnancy induced hypertension.

**Internal validation of prediction model for pregnancy induced hypertension.

**FIGURE 1:**
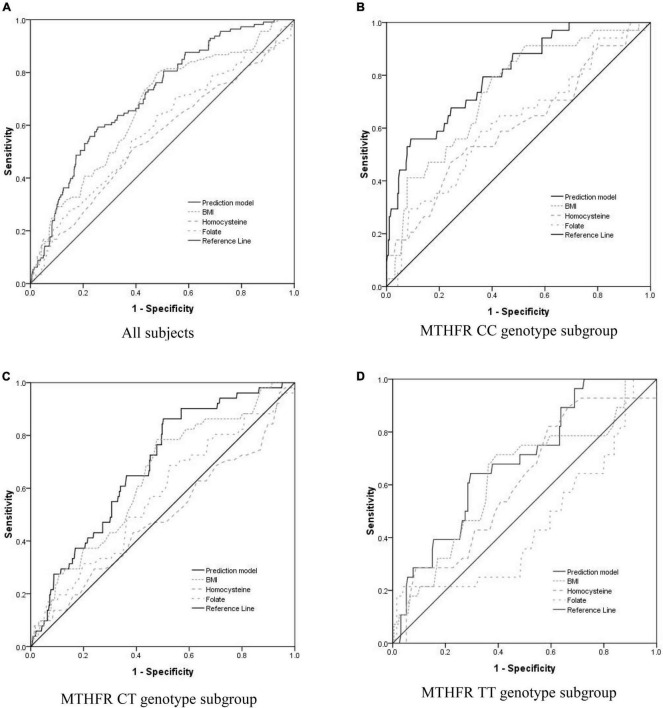
Evaluate the accuracy of the PIH prediction model including BMI and serum concentration of folate and homocysteine using receiver operating characteristic (ROC) curve analysis. **(A)** For All of the subjects, the area under the curve (AUC) of the prediction model, BMI, serum homocysteine concentration, and serum folate concentration was 0.711 (95% CI = 0.663–0.758, *p* < 0.0001), 0.664 (95% CI = 0.611–0.716, *p* < 0.0001), 0.554 (95% CI = 0.494–0.615, *p* = 0.059), and 0.580 (95% CI = 0.521–0.639, *p* = 0.006), respectively. **(B)** For the subgroup of subjects with MTHFR CC genotype, the AUC of the prediction model, BMI, serum homocysteine concentration, and serum folate concentration was 0.802 (95% CI = 0.725–0.878, *p* < 0.0001), 0.737 (95% CI = 0.652–0.821, *p* < 0.0001), 0.606 (95% CI = 0.498–0.713, *p* = 0.044), and 0.617 (95% CI = 0.513–0.720, *p* = 0.026), respectively. **(C)** For the subgroup of subjects with MTHFR CT genotype, the AUC of the prediction model, BMI, serum homocysteine concentration, and serum folate concentration was 0.684 (95% CI = 0.615–0.754, *p* < 0.0001), 0.638 (95% CI = 0.562–0.715, *p* = 0.001), 0.489 (95% CI = 0.398–0.579, *p* = 0.792), and 0.565 (95% CI = 0.479–0.651, *p* = 0.127), respectively. **(D)** For the subgroup of subjects with MTHFR TT genotype, the AUC of the prediction model, BMI, serum homocysteine concentration, and serum folate concentration was 0.685 (95% CI = 0.587–0.783, *p* = 0.002), 0.628 (95% CI = 0.514–0.742, *p* = 0.001), 0.609 (95% CI = 0.500–0.717, *p* = 0.063), and 0.449 (95% CI = 0.326–0.651, *p* = 0.573), respectively.

Receiver operating characteristic (ROC) curve analysis was utilized to evaluate the accuracy of a prediction model for PIH (Figure 1). For subjects of the normotensive group and the PIH group, the AUC of the prediction model was 0.711; the maximum value of its Youden index is 0.337; the sensitivity is 59.2%; the specificity is 74.4%. In the CC subgroup, the AUC of the prediction model was 0.802; the maximum value of its Youden index is 0.467; the sensitivity is 55.9%; the specificity is 90.8%. In the CT subgroup, the AUC of the prediction model was 0.684; the maximum value of its Youden index is 0.361; the sensitivity is 86.3%; the specificity is 49.8%. In the TT subgroup, the AUC of the prediction model was 0.685; the maximum value of its Youden index is 0.348; the sensitivity is 64.3%; the specificity is 70.5%. Therefore, the prediction model with homocysteine, folate, and BMI for PIH was more suitable for subjects with the MTHFR CC genotype.

### Association between MTHFR C677T genotype and serum levels of folate, homocysteine, BMI

**FIGURE 2:**
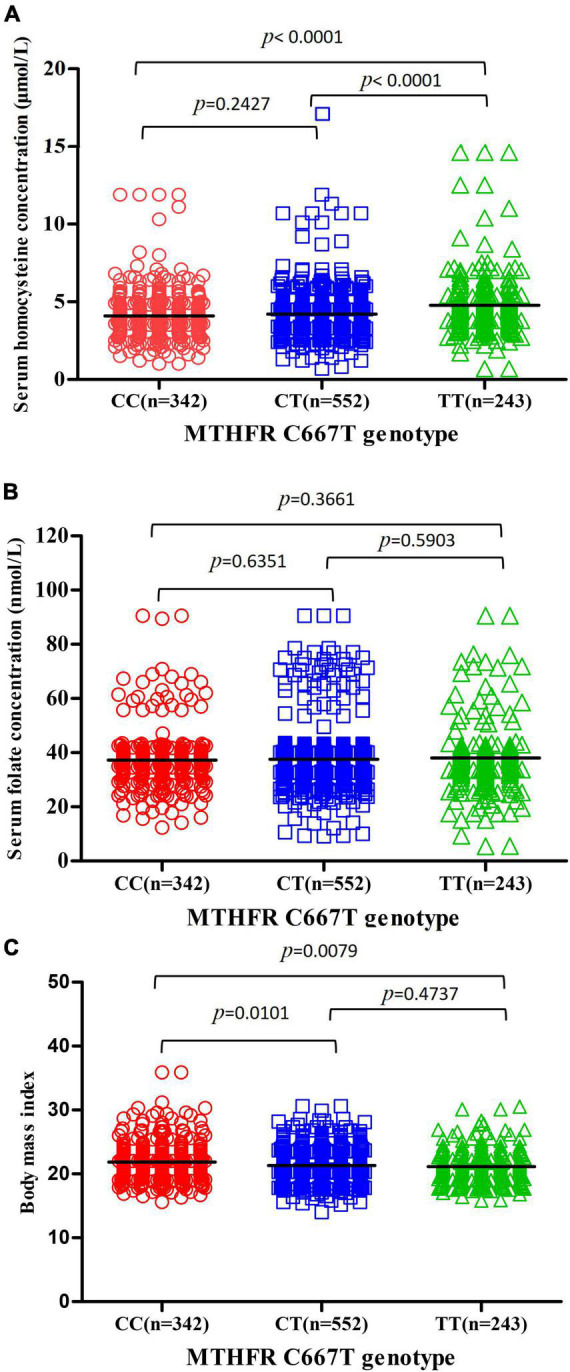
Association between MTHFR C677T genotype and serum homocysteine concentration, serum folate concentration, and BMI. **(A)** Association between MTHFR C677T genotype and serum homocysteine concentration. **(B)** Association between MTHFR C677T genotype and serum folate concentration. **(C)** Association between MTHFR C677T genotype and BMI.

**FIGURE 3:**
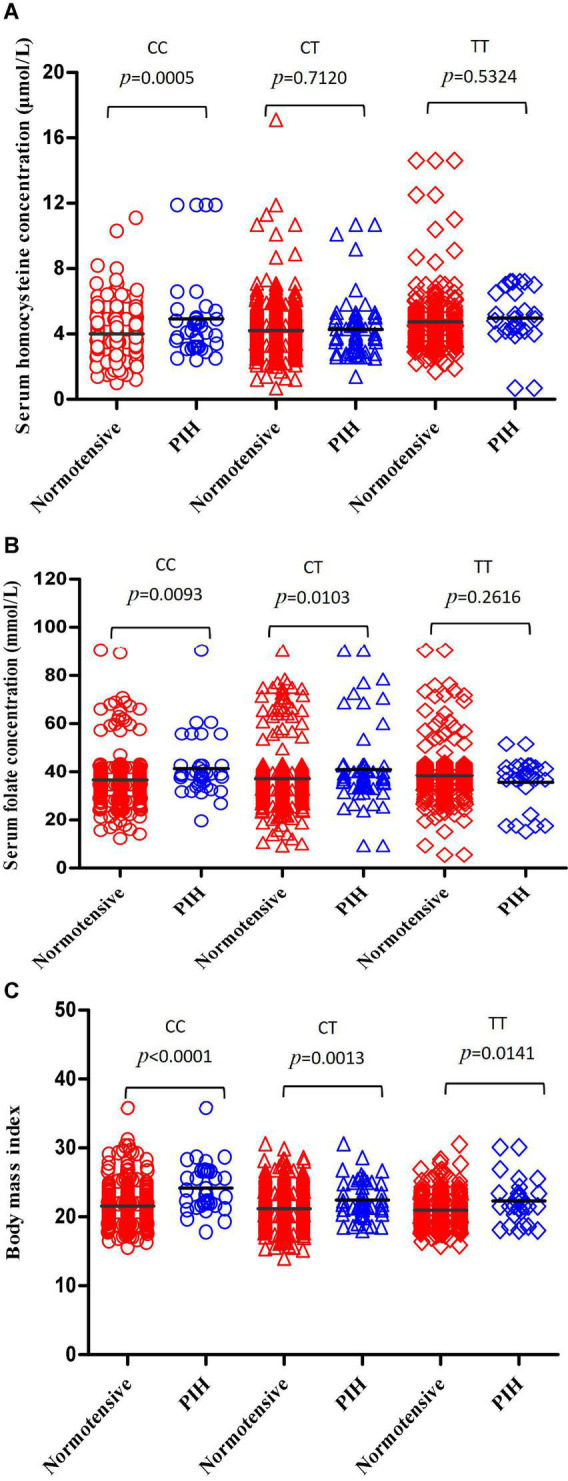
Compare the differences in serum homocysteine concentration, serum concentration, and BMI between the normotensive pregnancy group and PIH group in subgroups carrying different MTHFR C677T genotypes. **(A)** Compare the differences in serum homocysteine concentration between the normotensive pregnancy group and PIH group in subgroups carrying different MTHFR CC, CT, and TT genotypes, respectively. **(B)** Compare the differences in serum folate concentration between the normotensive pregnancy group and PIH group in subgroups carrying different MTHFR CC, CT, and TT genotypes, respectively. **(C)** Compare the differences in BMI between the normotensive pregnancy group and PIH group in subgroups carrying different MTHFR CC, CT, and TT genotype, respectively.

Serum homocysteine levels of subjects with MTHFR CC, CT, and TT genotypes were 4.090 ± 1.545, 4.210 ± 1.503, and 4.773 ± 1.903 μmol/L, respectively, and there was a significant difference among the three subgroups (*p* < 0.0001) (Figure 2A). Serum homocysteine levels of the PIH group were higher than that of the normotensive group in the CC subgroup (*p* = 0.0005); no significant difference in homocysteine levels was observed in the CT and TT subgroup (*p* = 0.7120, 0.5324, respectively) (Figure 3A).

Serum folate levels of subjects with MTHFR CC, CT, and TT genotypes were 37.20 ± 10.36, 37.56 ± 11.34, and 38.04 ± 11.87 mmol/L, respectively, and there was no significant difference among the three subgroups (*p* = 0.3661) (Figure 2B). Serum folate levels of the PIH group were higher than that of the normotensive group in the CC and CT subgroup (*p* = 0.0093, 0.0103, respectively); no significant difference in folate levels was observed in the TT subgroup (*p* = 0.2616) (Figure 3B).

The body mass index of subjects with MTHFR CC, CT, and TT genotypes were 21.70 ± 3.540, 21.31 ± 2.668, and 20.97 ± 3.305, respectively, and there were significant differences among three subgroups (*p* = 0.0079) (Figure 2C). The BMI of the PIH group was higher than that of the normotensive group in all three subgroups (*p* < 0.0001, *p* = 0.0013, and 0.0141, respectively) (Figure 3C).

### Correlations between vitamin B12, homocysteine, folate, and BMI in subjects with different MTHFR C677T genotype

A negative correlation between BMI and serum vitamin B12 concentration appeared in both CC and CT + TT subgroups QQ(*p* = 0.008, < 0.001, respectively). A positive correlation between vitamin B12 and folate level was found in both CC and CT + TT subgroups (*p* = 0.047, 0.001, respectively). A negative correlation between folate and homocysteine concentration only was observed in the CT + TT subgroup (*p* = 0.005), while there was no correlation between folate and homocysteine concentration in the CC subgroup (*p* = 0.215) (Supplementary Table 1).

### Establishment prediction model for PE in pregnant women with PIH

Respective impact of 10 potential risk factors including age, weight, BMI, number of embryos, gravidity, parity, serum levels of vitamin B12, folate, homocysteine, and MTHFR C677T genotype, on the incidence of PE were assessed by binary logistic linear regression analysis based on Enter method for the variable introduction. The number of embryos (*p* = 0.005, OR = 6.369, 95% CI = 1.728–23.471) was a statistically significant risk factor for PE; while gravidity (*p* = 0.012, OR = 0.413, 95% CI = 0.207–0.822), serum folate concentration (*p* = 0.028, OR = 0.944, 95% CI = 0.896–0.994) were statistically significant protective factors for PE (Supplementary Table 2).

The AUC of the prediction model for PE including the number of embryos, gravidity, and serum folate concentration was 0.781 (*p* < 0.0001) using ROC curve analysis. The maximum value of its Youden index is 0.536, the sensitivity is 97%, and the specificity is 56.6% (Supplementary Figure 1).

## Discussion

Real world data of 1,178 pregnant women undergoing genotyping of MTHFR C677T and supplementation of folate and vitamin B12 in our hospital were incorporated into this study. OCM-related indicators presented high folate, median vitamin B12, and low homocysteine status; the gene distribution of MTHFR C677T was consistent with previous studies performed in the Chinese Han population (13). A total of 11.12% of subjects (131 cases) suffered from PIH; the incidence rate of PE was 3.48% (41 cases). The general information [age, weight, body mass index (BMI), number of embryos, gravidity, and parity] and OCM-related indicators (serum levels of vitamin B12, folate, homocysteine, and MTHFR C677T genotype) were included in the logistic linear regression analysis for screening the risk factor of PIH. A model consisting of serum homocysteine, folate concentration, and BMI was established for predicting PIH. In a study on the modeling of new-onset hypertension in the Chinese Han population, 22 indexes, including general information, complete blood count, biochemical test, and homocysteine were analyzed as potential risk factors of PIH. Seven indexes were screened as independent risk factors by binary logistic regression and the equation was as follows: *Y* = (−5.855) + 0.590 (cystatin C)–0.766 (globulin) + 0.591 (uric acid) + 0.399 (BMI) + 0.305 (age) + 0.332 (homocysteine) + 0.526 (total bile acid) (16). Homocysteine and BMI were also incorporated into the model for predicting new-onset hypertension. Our results were in accordance with this study. However, other OCM-related indicators which might influence homocysteine levels were not analyzed in this study. As we know, folate and vitamin B12 were major participants of OCM and the supplementation of folate and vitamin B12 might change the interrelation among OCM-related indicators and their effects on PIH incidence. In the present study, we first explored the prediction model for PIH using OCM-related indicators in the Chinese Han population with supplementation of folate and vitamin B12.

The impact of high serum homocysteine, folate level, and BMI on PIH incidence had been well documented. Hyperhomocysteine was a key risk factor for hypertension in the Chinese Han population. A case-control study enrolled 59 cases of PIH and 60 cases of normal late pregnancy and showed that serum homocysteine levels of the PIH group were significantly higher than that of the normal pregnancy group and there were no statistical differences in the level of folate and vitamin B12 between the two groups (17). This suggested that homocysteine metabolism might play an important role in the pathological mechanism of PIH; there was no direct evidence that folate and vitamin B12 were related to PIH. Cases of this study did not receive the intervention of folate and vitamin B12 supplements. High serum folate levels were associated with PIH incidence in univariate and multivariate analyses in our study. Jankovic-Karasoulos et al. (7) demonstrated that pregnant women supplemented with folate increased circulating folate and vitamin B12 levels compared with those who did not supplement; and serum folate levels were higher in women who developed gestational hypertension (GHT) and gestational diabetes mellitus (GDM) compared with those with uncomplicated pregnancies. Liu et al. (18) probed into the relationship between prepregnancy BMI and the risk of PIH in a large sample study that included 83,159 Chinese pregnant women. The PIH incidence was 11.01%. The incidence of PIH among pregnant women with BMI <18.5, 18.5–22.9, 23.0–24.9, and ≥25.0 kg/m^2^ were 9.08, 10.82, 14.63, and 19.00%, respectively, the difference was significant. High prepregnancy BMI might increase the risk of developing PIH in pregnant women.

Methylenetetrahydrofolate reductase was an essential enzyme that influenced the relationship between folate intake and homocysteine serum level. MTHFR C677T polymorphism (rs 1801133) was the most commonly reported genotype associated with the phenotype of homocysteine serum level. A base substitution (C→T) at position 677 of the MTHFR gene resulted in an alanine to valine substitution and reduced enzyme activity, which led to low folate concentration and high homocysteine concentration in serum, especially in the circumstances of folate insufficiency (11, 19). In the present study, there was no significant difference in the incidence of PIH among the three groups with MTHFR C677T CC, CT, and TT genotypes, and the MTHFR C677T genotype was not the risk factor of PIH based on logistic linear regression analysis. The associations of the MTHFR C677T genotype with PIH remained controversial. A meta-analysis from 114 studies with 15,411 cases and 21,970 controls showed that the C677T polymorphism was significantly associated with PIH in the overall subjects and hierarchical analysis by ethnicity revealed that this association existed in Asians and Caucasians, but not in Latinos, Black Africans, and Indians and Sri Lankans (13). Hernández-Díaz et al. (20) compared the prevalence of the 677TT/CT genotypes between 54 cases with PIH and 100 controls with normal blood pressure in the US and Canadian white women population. This study found that maternal MTHFR C677T polymorphism may be associated with a moderately increased risk of PIH, and this association may be diminished among women supplemented with folic acid during the first 5 months of pregnancy. No association between MTHFR C677T genotype and PIH incidence in our study might due partly to suitable supplementation of folate.

Furthermore, the hierarchical analysis according to the MTHFR C677T genotype was made to observe the effect of OCM-related indicators and BMI on PIH. The accuracy of the prediction model with homocysteine, folate, and BMI for PIH in subjects with MTHFR CC genotype (AUC: 0.802) was obviously higher than that in subjects with MTHFR CT, TT genotype (AUC: 0.684,0.685, respectively). MTHFR C677T genotype had a complex influence on all three factors in the prediction model. Kim et al. (21) explored the effects of the interaction between MTHFR polymorphism and serum B vitamins on homocysteine levels in pregnant women without medication intervention. Serum homocysteine level was higher in women with the MTHFR TT genotype than those with MTHFR CT and TT genotype. A negative correlation between serum homocysteine and folate was observed in all MTHFR genotypes, especially in women with the TT genotype. Serum folate level and MTHFR C677T genotype were the determinants for homocysteine levels (22). Association between MTHFR gene polymorphisms and BMI had been confirmed in several studies (23). Su et al. (24) found a predominant association between MTHFR C677T and weight gain after risperidone antipsychotic treatment. After a 6-week treatment of risperidone treatment, the BMI increase rate of subjects with the MTHFR CC, CT genotype was significantly higher than that of the MTHFR TT genotype. The impact of the MTHFR C677T genotype on serum homocysteine level and BMI has also been observed in our study, indicating that all three factors in the model had a significant predictive value of PIH only in women who carried the CC genotype.

We investigated the correlations between vitamin B12, homocysteine, folate, and BMI in subjects with different MTHFR C677T genotypes, which might be the characteristic features of pregnant women supplemented with folate and vitamin B12 and affected the occurrence of PIH. A negative correlation between folate and homocysteine concentration only was observed in the CT + TT subgroup. Folate intake and serum folate level were the main factors influencing homocysteine concentration besides genetic, health, and lifestyle factors in the general population and pregnant women. Chaudhry et al. (25) investigated influencing factors of the homocysteine level of pregnant women, particularly in a folate-supplemented population. Folate concentration was linearly correlated with the dose when the folate supplement dose is less than 1 mg. There was no correlation between serum folate and serum homocysteine in subjects with CC/CT genotypes, whereas serum folate and serum homocysteine demonstrated a steep negative slope-shaped association in subjects with TT genotypes. Therefore, it can be inferred that MTHFR 677C > T genotype modified the effect of serum folate on serum homocysteine in the folate-supplemented pregnant women.

Although a positive correlation between vitamin B12 and folate levels was found in both CC and CT + TT subgroups of our research object, the PIH group had high folate and low vitamin B12 levels compared to the normotensive pregnancy group. A UK prospective multicenter study (*n* = 4,746) showed that B12 insufficiency and folate excess were common in early pregnancy. Folate supplementation led to high unmetabolized folate serum levels. Unmetabolized folate increased intracellular ATP accumulation and reduced glucose uptake by muscle cells mediated by insulin, which resulted in higher insulin resistance. Insulin resistance was closely related to hypertension (26). A high folate/low B12 led to a functional folate deficiency and impaired re-methylation of homocysteine. Vitamin B12 deficiency could also aggravate insulin resistance by impairing the conversion of methylmalonyl-CoA to succinyl-CoA (27). Thus, high folate/low B12 caused PIH through a variety of mechanisms.

Positive feedback interaction between Vitamin B12 and BMI had been reported. Allin et al. (28) found an association between decreased serum vitamin B12 and increased BMI, and a 20% decrease in serum vitamin B12 brought about a 0.09 kg/m^2^ increase in BMI by instrumental variable regression. FUT2 might partially explain the effect of serum vitamin B12 on BMI through its regulation of the interaction between intestinal flora and host. A meta-analysis demonstrated a possible significant inverse correlation between prepregnancy BMI and levels of micronutrients, including vitamin B12 (29). A negative correlation between BMI and vitamin B12 also appeared in the present study. Although no significant effect of vitamin B12 on PIH was observed in our data, vitamin B12 is also an important participant in the pathological process by statistical or biological correlation of folate, BMI, and insulin resistance. The impact of vitamin B12 on PIH incidence should be explored in a further prospective randomized controlled study.

According to some reports and our data, the most extraordinary discovery in pregnant women with folate and vitamin B12 supplementation of our study was that model with homocysteine, folate, and BMI for PIH was available to predict the incidence of PIH in subjects with MTHFR CC genotype (AUC: 0.802), but not in subjects with MTHFR CT and TT genotype (AUC < 0.7). Our hypothesis for the results observed was that folate and vitamin B12 supplementation could change the original association mode between OCM-related indicators, which impact the incidence of PIH: the association between MTHFR C677T polymorphism and PIH might be diminished by folate supplementation. The disappearance of the impact of OCM-related indicators on PIH in the CT + TT subgroup could be that high serum folate and homocysteine levels were both associated with PIH incidence and a negative correlation between folate and homocysteine concentration was observed in the CT + TT subgroup in pregnant women with folate supplementation; a positive correlation between vitamin B12 and folate level was found in both CC and CT + TT subgroups of our research object.

Preeclampsia was a serious stage of HDP and increased risk of maternal and neonatal mortality. Also, women with a PE history had an increased risk of cardiovascular disease later in life compared with normotensive pregnant women. Preventing the incidence of PE in women with PIH could be an effective measure to reduce the harm of HDP. Our data demonstrate that multiple embryos, nulliparas, and lower folate serum levels were risk factors in the developing process from PIH to PE based on logistic linear regression analysis. Multiple births and first pregnancy were generally recognized to increase the risk of preeclampsia (30, 31). Two randomized clinical trials confirmed that early (3 months before pregnancy) and high dose (4.0 mg/day) folate supplement reduced recurrent PE, and high dose folate supplementation in later pregnancy (beyond the first trimester) could not prevent PE (32, 33). Vitamin B12 decreased gradually in the normotensive pregnancy group, the PIH group, and the PE group. Kharb et al. (34) suggested that vitamin B12 deficiency might be a risk factor for PE and future risk of cardiovascular risk.

The main strength of our study was to explore the impact of OCM pathway-related indicators on pregnancy-induced hypertension diseases using real-world data. There was a great difference in the number of cases between the normotensive pregnancy group (*n* = 1,006) and the PIH group (*n* = 131). The incidence rate of PIH (11.12%) was close to real-world clinical practice. In this event, the matched-group design could lead to uncontrollable bias for this type of research (35). All data of the normotensive pregnancy group were involved in the result analysis in the present study. The results of this study were from actual clinical situations and were ready to be applied.

This study had several limitations. First, the impact of single OCM-related indicators on PIH and PE in pregnant women supplemented with vitamin B12 and folate should be further confirmed by prospective, randomized, and controlled studies. Second, multicenter data should be collected to clarify the extrapolation of this study’s experimental conclusions. Third, mechanism research of changes in OCM mediated by folate and vitamin B12 should be made in future research.

In conclusion, there were complex interactions between related indicators of OCM, including serum levels of vitamin B12, folate, homocysteine, and MTHFR C677T genotype. The prediction model composed of homocysteine, folate, and BMI for PIH was more suitable for subjects with MTHFR CC genotype in pregnant women with supplementation of folate and vitamin B12. Lower folate levels could be an independent risk factor in developing the process from PIH to PE.

## Data availability statement

The original contributions presented in this study are included in the article/Supplementary materials, further inquiries can be directed to the corresponding authors.

## Ethics statement

The studies involving human participants were reviewed and approved by Ethics Committee of IPMCH. The patients/participants provided their written informed consent to participate in this study.

## Author contributions

XZ and YW designed this research. YZ, CG, YL, JW, and LS collected the clinical data. XZ and YZ interpreted data. JF performed statistical analysis and mapping. XZ and JF wrote the manuscripts. All authors contributed to editing and reviewing the manuscript.
