# Supplementary material for: Interrelation among one-carbon metabolic (OCM) pathway-related indicators and its impact on the occurence of pregnancy-induced hypertension disease in pregnant women supplemented with folate and vitamin B12: Real-world data analysis
Source: Front Nutr. 2023 Jan 10;9:950014. doi: 10.3389/fnut.2022.950014 (PMC9871780; doi:10.3389/fnut.2022.950014)
Supplement: Supplementary file 1 [file Data_Sheet_1.docx]

| **Supplementary table 1.** Correlations between vitamin B12, homocysteine, folate, and BMI in subjects with different MTHFR C677T genotype | | | | | | |
| --- | --- | --- | --- | --- | --- | --- |
|  |  |  | Body mass index | vitamin B12 concentration | folate concentration | homocysteine concentration |
| MTHFR C677T CC genotype subgroup | Body mass index | Pearson Correlation | 1 | -.147** | -.068 | -.027 |
|  |  | Sig. (2-tailed) |  | .008 | .225 | .632 |
|  | vitamin B12 concentration | Pearson Correlation | -.147** | 1 | .109* | -.060 |
|  |  | Sig. (2-tailed) | .008 |  | .047 | .274 |
|  | Folate concentration | Pearson Correlation | -.068 | .109* | 1 | .067 |
|  |  | Sig. (2-tailed) | .225 | .047 |  | .215 |
|  | homocysteine concentration | Pearson Correlation | -.027 | -.060 | .067 | 1 |
|  |  | Sig. (2-tailed) | .632 | .274 | .215 |  |
| MTHFR C677T CT+TT genotype subgroup | Body mass index | Pearson Correlation | 1 | -.234 | .000 | -.047 |
|  |  | Sig. (2-tailed) |  | .000 | .986 | .209 |
|  | vitamin B12 concentration | Pearson Correlation | -.234 | 1 | .115 | -.013 |
|  |  | Sig. (2-tailed) | .000 |  | .001 | .712 |
|  | folate concentration | Pearson Correlation | .000 | .115 | 1 | -.099 |
|  |  | Sig. (2-tailed) | .986 | .001 |  | .005 |
|  | homocysteine concentration | Pearson Correlation | -.047 | -.013 | -.099 | 1 |
|  |  | Sig. (2-tailed) | .209 | .712 | .005 |  |

| **Supplementary table 2.** Effect of general information and OCM related indicators on the progression of PIH to PE (n=172) * | | | | | | | |
| --- | --- | --- | --- | --- | --- | --- | --- |
|  | B | S.E. | Wald | Sig. | Exp(B) | 95.0% C.I.for EXP(B) | |
| variables |  |  |  |  |  | Lower | Upper |
| Age | .039 | .067 | .344 | .558 | 1.040 | .912 | 1.186 |
| weight | -.106 | .056 | 3.626 | .057 | .900 | .807 | 1.003 |
| Body mass index | .093 | .146 | .403 | .526 | 1.097 | .824 | 1.460 |
| Number of embryos | 1.851 | .665 | 7.740 | .005 | 6.369 | 1.728 | 23.471 |
| Gravidity | -.885 | .351 | 6.345 | .012 | .413 | .207 | .822 |
| Parity | .780 | .603 | 1.674 | .196 | 2.182 | .669 | 7.113 |
| MTHFR C677T genotype | .184 | .324 | .325 | .569 | 1.202 | .638 | 2.267 |
| Serum vitamin B12 concentration (ng/L) | -.001 | .002 | .495 | .482 | .999 | .995 | 1.002 |
| Serum homocysteine concentration (µmol/L) | .012 | .120 | .011 | .918 | 1.012 | .800 | 1.282 |
| Serum folate concentration (mmol/L) | -.058 | .026 | 4.845 | .028 | .944 | .896 | .994 |
| Constant | 3.285 | 3.155 | 1.085 | .298 | 26.717 |  |  |


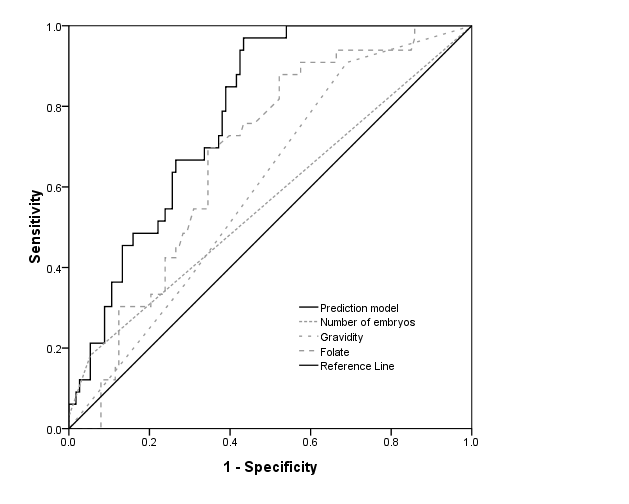


**Supplementary Figure 1**
